# Identification of a Shared Cytochrome p4502E1 Epitope Found in Anesthetic Drug-Induced and Viral Hepatitis

**DOI:** 10.1128/mSphere.00453-18

**Published:** 2018-10-10

**Authors:** Elisa K. McCarthy, Amanda Vakos, Merylin Cottagiri, Joel J. Mantila, Lakshmi Santhanam, David L. Thomas, L. Mario Amzel, Noel R. Rose, Dolores B. Njoku

**Affiliations:** a Department of Anesthesiology and Critical Care Medicine, Johns Hopkins University, Baltimore, Maryland, USA; b Division of Infectious Disease, Department of Medicine, Johns Hopkins University, Baltimore, Maryland, USA; c Department of Biophysics and Biophysical Chemistry, Johns Hopkins University, Baltimore, Maryland, USA; d Department of Pathology, Brigham and Women’s Hospital, Harvard University, Cambridge, Massachusetts, USA; e Department of Pathology, Johns Hopkins University, Baltimore, Maryland, USA; National Institute of Allergy and Infectious Diseases

**Keywords:** autoantibody, mitochondria complex 1, oxidative stress

## Abstract

Cytochrome p4502E1 (CYP2E1) autoantibodies are biomarkers for drug-induced hepatitis and chronic hepatitis C. However, major histocompatibility-restricted CYP2E1 epitopes associated with these diseases have not been identified. We hypothesized that CYP2E1 epitopes associated with different types of hepatitis may be shared and may impact immune responses and metabolism. SYFPEITHI epitope prediction identified CYP2E1 candidate epitopes that would be recognized by MHC II haplotypes. Candidate epitopes were tested for induction of hepatitis and CYP2E1 autoantibodies in mice and recognition by sera from patients with anesthetic drug-induced and viral hepatitis. Human liver cells treated with epitope hybridoma serum were analyzed for mitochondrial stress. CYP2E1 activity was measured in human microsomes similarly treated. Epitope antibodies in viral hepatitis sera were analyzed using linear regression to uncover associations with liver pathology. A *P* value of <0.05 was considered significant. One epitope (Gly^113^-Leu^135^) induced hepatitis and CYP2E1 autoantibodies in mice after modification of Lys^123^ (*P < *0.05). Gly^113^-Leu^135^ antiserum recognized mitochondria and endoplasmic reticula (*P < *0.05), upregulated HSP27 (*P* < 0.01) and mitochondrial oxidative stress via complex 1 inhibition (*P* < 0.001), and inhibited CYP2E1 activity. Gly^113^-Leu^135^ IgG4 detected in viral hepatitis sera was associated with severe hepatic fibrosis (*P* = 0.0142). We found a novel CYP2E1 epitope that was detected in anesthetic and viral hepatitis and that triggered hepatitis in mice. Our findings may improve understanding of hepatic immune responses triggered by metabolism or viruses.

**IMPORTANCE** Drug-induced hepatitis is the leading reason that an approved drug is removed from the commercial market. Halogenated anesthetics can induce hepatitis in susceptible persons, and cytochrome p4502E1 (CYP2E1) enzymes responsible for their metabolism induce antibodies in addition to hepatitis. CYP2E1 antibodies detected in anesthetic hepatitis patients have been detected in patients with viral hepatitis, suggesting that these different forms of hepatitis could develop immune reactions to a common segment or epitope of CYP2E1. We have found a common MHC-restricted CYP2E1 epitope in anesthetic and viral hepatitis that is a dominant epitope in anesthetic hepatitis and is significantly associated with fibrosis in patients with viral hepatitis. Along with conformational epitopes, our identification of MHC-restricted CYP2E1 epitopes can be used to develop specific diagnostic tests for drug-induced or viral hepatitis or associated fibrosis or to predict individuals at risk for developing these diseases or their sequelae.

## Abstract (precis)

Drug-induced hepatitis is the leading reason that an approved drug is removed from the commercial market. Halogenated anesthetics can induce hepatitis in susceptible persons, and cytochrome p4502E1 (CYP2E1) enzymes responsible for their metabolism induce antibodies in addition to hepatitis. CYP2E1 antibodies detected in anesthetic hepatitis patients have been detected in patients with viral hepatitis, suggesting that these different forms of hepatitis could develop immune reactions to a common segment or epitope of CYP2E1. We have found a common MHC-restricted CYP2E1 epitope in anesthetic and viral hepatitis that is a dominant epitope in anesthetic hepatitis and is significantly associated with fibrosis in patients with viral hepatitis. Along with conformational epitopes, our identification of MHC-restricted CYP2E1 epitopes can be used to develop specific diagnostic tests for drug-induced or viral hepatitis or associated fibrosis or to predict individuals at risk for developing these diseases or their sequelae.

## INTRODUCTION

Cytochrome p4502E1 (CYP2E1) is a key player in hepatic drug metabolism (1) and is responsible for the oxidative metabolism of halogenated anesthetics. Following oxidative metabolism by CYP2E1, toxic (2) or immune-mediated hepatitis (3) as well as formation of CYP2E1 autoantibodies (4) occusr in susceptible patients. CYP2E1 autoantibodies are a biomarker for immune-mediated anesthetic hepatitis (3, 4) and halothane toxicity (5, 6), but there is a long-held belief that these autoantibodies are by-products of halogenated anesthetic exposure without consequences. Hence, epitopes of CYP2E1 autoantibodies and their subsequent immune or metabolic responses have not been well studied.

Interleukin-4 (IL-4) is connected to CYP2E1 and anesthetic hepatitis. IL-4 upregulates transcription of CYP2E1 via Janus kinase-signal transducer and activator of transcription 6 and transcription factor nuclear factor of activated T cells, cytoplasmic 1 induction of insulin receptor substrate 1/2 (7, 8). IL-4 initiates anesthetic hepatitis, its associated CYP2E1 autoantibodies (9), and toxic halothane hepatitis (10). However, CYP2E1 epitopes that trigger autoantibodies or hepatitis are unknown, and their role in disease pathogenesis is unclear. Even so, it is important to identify CYP2E1 epitopes because these epitopes can be utilized to develop specific diagnostic tests or to predict individuals at risk for developing drug or virus-induced hepatitis.

Posttranslational modification of lysine in hepatic proteins, including CYP2E1, is implicated in the pathogenesis of anesthetic hepatitis and may also induce CYP2E1 autoantibodies (11). Hence it is currently accepted that anesthetic hepatitis is triggered by neoantigens produced when liver proteins such as CYP2E1 become covalently modified by trifluoroacetyl chloride (TFA) drug metabolites formed during anesthetic oxidative metabolism by CYP2E1 (11). We have modeled this mechanism in BALB/c mice, making them susceptible to the development of hepatitis and production of autoantibodies (3). Reduced hepatitis and CYP2E1 autoantibodies in IL-4-deficient mice, as well as the detection of CYP2E1 IgG4 subclass autoantibodies in patients with anesthetic hepatitis, support a role for IL-4 in the development of CYP2E1 autoantibodies and hepatitis (4, 9, 12). Even so, without knowing CYP2E1 immunogenic epitopes, the significance of posttranslational modification of CYP2E1 in hepatitis or CYP2E1 autoantibodies may be underestimated.

CYP2E1 autoantibodies are not unique to anesthetic hepatitis. They have been detected in hepatitis from alcohol (13) and hepatitis C virus (HCV) (14). In chronic hepatitis C (CHC), CYP2E1 autoantibodies are a biomarker for necroinflammation (15), which may suggest functional roles for these autoantibodies. Interestingly, molecular simulation and single amino acid mutagenesis have been used to predict CYP2E1 epitopes in a sample of halothane and alcoholic hepatitis patients (16), while prior studies have not been able to detect CYP2E1 epitopes in the context of major histocompatibility complex (MHC) restriction. Even so, detecting CYP2E1 autoantibodies in all three forms of hepatitis suggests that a common CYP2E1 epitope may be responsible.

CYP2E1 is connected to reactive oxygen species (ROS). CYP2E1 enzymatic functions promote hepatic oxidative stress by generating ROS (17). Lipid peroxidation and heat shock proteins (HSPs) also participate in the generation of ROS (18). The importance of ROS is well established in alcoholic hepatitis (19) and drug-induced liver injury (DILI) from acetaminophen (20). In addition, HCV core proteins may promote mitochondrial oxidative stress (21, 22). However, although CYP2E1 has a key role in the pathogenesis of drug-induced hepatitis following anesthetics, roles for ROS in the pathogenesis of disease have not been previously described.

We have identified one CYP2E1 epitope, glycine^113^-leucine^135^ (Gly^113^-Leu^135^), that is recognized by mice and by sera from patients with anesthetic or viral hepatitis. In BALB/c mice, Gly^113^-Leu^135^ triggers hepatitis as well as CYP2E1 antibodies after modification of Lysine^123^ (Lys^123^) with TFA. We show that Gly^113^-Leu^135^ antiserum colocalizes with mitochondria and endoplasmic reticulum, inhibits CYP2E1 enzyme activity in human microsomes, increases mitochondrial oxidative stress via complex 1 inhibition, and upregulates ROS-responsive HSP27 *in vitro*. Elevated Gly^113^-Leu^135^ IgG4 subclass autoantibodies detect anesthetic hepatitis and associate with severe hepatic fibrosis in patients with viral hepatitis. Our studies highlight a common MHC-restricted CYP2E1 epitope in anesthetic and viral hepatitis with immune and metabolic consequences and strongly suggest that this epitope could be the dominant CYP2E1 epitope in anesthetic hepatitis.

## RESULTS

### Candidate epitopes.

BALB/c mice express major histocompatibility (MHC) II haplotypes I-Ad and I-Ed and are uniquely susceptible to experimental anesthetic hepatitis, where they generate cytochrome p4502E1 (CYP2E1) autoantibodies (3, 9). We generated 30 CYP2E1 candidate epitopes for each mouse haplotype using the RANKPEP prediction of peptide binding to class II MHC molecules (23). Human CYP2E1 was used to generate epitopes because a large number of candidate epitopes were identical between human and mouse CYP2E1 (18 I-Ad and 13 I-Ed candidate epitopes). Four candidate epitopes were selected from the top two epitopes generated for each haplotype (see Table S1A in the supplemental material, JHDN-1 to -4).

10.1128/mSphere.00453-18.5 TABLE S1 (A) Candidate epitopes generated with the RANKPEP software program. (B) Candidate epitopes centered on the CYP2E1 active site. Download Table S1, PDF file, 0.01 MB. Copyright © 2018 McCarthy et al. 2018 McCarthy et al. This content is distributed under the terms of the Creative Commons Attribution 4.0 International license.

A prior study showed that CYP2E1 autoantibody-positive sera from halothane hepatitis patients inhibited CYP2E1 enzymatic activity in microsomes *in vitro* (5), suggesting to us that a critical CYP2E1 epitope may be proximal to the CYP2E1 active site. However, none of the RANKPEP-generated epitopes were proximal to the CYP2E1 active site. Consequently, we sequenced five additional 18- to 20-mer CYP2E1 candidate epitopes around Ser^129^, the CYP2E1 active site (Table S1B, JHDN-5 to -9). Negative charges were conferred to candidate epitopes by blocking and charging cysteine residues with amidation and acetylation in an attempt to improve recognition in the MHC II binding groove (24). A total of nine candidate epitopes were generated.

### DO11.10 I-Ad T cell assays recognized four CYP2E1 candidate epitopes.

**FIG 1:**
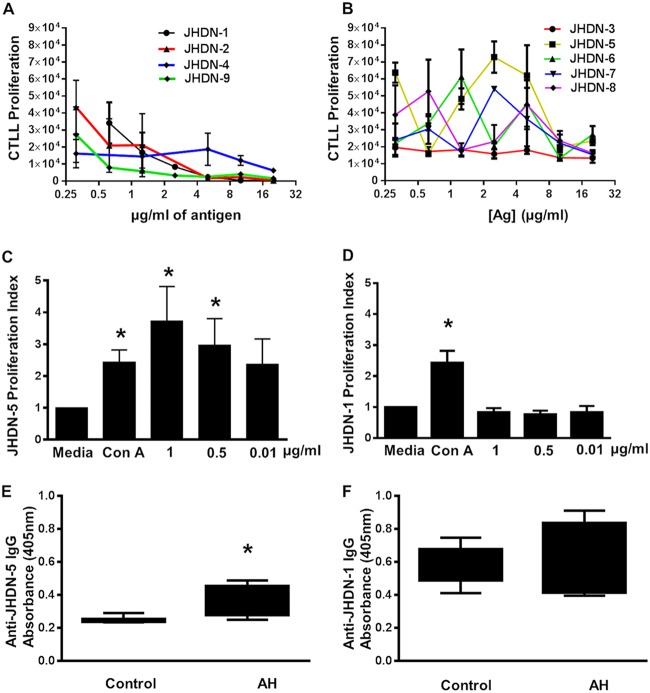
Identification of CYP2E1 epitopes. (A and B) Candidate CYP2E1 epitopes were evaluated for I-Ad recognition using DO11.10 T cell hybridomas cultured with 0.5 mg ovalbumin^323–329^. Proliferation of CTLL-2 cells measured by [^3^H]thymidine incorporation confirmed IL-2 production. Mouse I-Ad recognized JHDN 1, 2, 4, and 9 but not JHDN 3, 5, 6, and 8. (C and D) BALB/c splenocytes 2 weeks after immunization with TFA-S100 (days 0 and 7) were challenged with JHDN-5 (10 µg/ml), JHDN-1 (10 µg/ml), or ConA (1 µg/ml) *in vitro*. [^3^H]thymidine incorporation after 48 h demonstrated JHDN-5 recognition (C) (*, *P* < 0.05) but not JHDN-1 (D). (E and F) Human sera (1:100) from patients with anesthetic DILI (AH, *n =* 44) and controls (*n =* 45) were tested for antibodies to JHDN-1 and JHDN-5 (0.5 µg/100 µl) by ELISA (405 nm) using AKP-IgG secondary antibodies. AH patients had increased levels of JHDN-5 IgG (E) (*, *P* < 0.05) but not JHDN-1 IgG (F) compared to controls.

DO11.10 I-Ad T cell competitive inhibition assays recognized JHDN 1, 2, 4, and 9 (Fig. 1A), but not JHDN 3, 5, 6, 7, and 8 (Fig. 1B). These results suggested that three of the candidate epitopes generated by the RANKPEP and one of the CYP2E1 Ser^129^ active site may be responsible for CYP2E1 autoantibodies and possibly anesthetic hepatitis.

### JHDN-5 may be recognized by I-Ed.

Because we were unable to locate an I-Ed T cell hybridoma for analysis, we tested candidate epitopes for recognition by splenocytes isolated from our drug-induced hepatitis model in BALB/c mice (3). We had previously shown that the model develops hepatitis and CYP2E1 autoantibodies (9). We found that splenocytes from BALB/c mice immunized with trifluoroacetyl chloride (TFA)-altered liver proteins recognized JHDN-5 (Fig. 1C) but not JHDN-1 (Fig. 1D) or any other epitope. These results suggest that JHDN-5 is recognized in drug-induced hepatitis and is likely recognized by I-Ed.

To clarify if JHDN-5 is preferentially recognized by I-Ed, we used SYFPEITHI epitope prediction to first compare binding scores of JHDN-5 with those for ovalbumin (OVA)^323–339^, a known I-Ad peptide. We found a SYFPEITHI score of 15 for OVA and a score of −2 for JHDN-5 (Table 1). We then compared binding scores of JHDN-5 with those for hen egg white lysozyme (HEL)^107–116^, a known I-Ed peptide, and found that the score for both was 18 (Table 1). Thus, our findings support our idea that JHDN-5 is a promising CYP2E1 epitope in drug-induced hepatitis and is recognized by I-Ed.

**TABLE 1:** SYFPEITHI prediction score comparisons for JHDN5 and known I-Ad and I-Ed peptides

Epitope^*a*^	MHC II haplotype	Sequence	SYFPEITHI score
OVA^323–339^	I-Ad	ISQAVHAAHAEINEAGR	18
JHDN5	I-Ad	GIIFNNGPTKDIRRFSLTTL	−2
HEL^107–116^	I-Ed	MNAWVAWRKRCKGTDV	18
JHDN5	I-Ed	GIIFNNGPTKDIRRFSLTTL	18

a OVA, ovalbumin; HEL, hen egg white lysozyme.

### JHDN-5 is recognized by sera from patients with drug-induced hepatitis from halogenated anesthetics.

To address the possibility that mouse or SYFPEITHI epitope recognition may not translate to human disease, we tested candidate CYP2E1 epitopes for recognition by sera from anesthetic hepatitis (AH) patients. We accepted the possibility that a separate B cell epitope may be identified in patients that may not coincide with a T cell epitope in mice.

Significantly higher levels of autoantibodies to JHDN-5 were detected in the sera of AH than in that of control patients (Fig. 1E). Similar to our mouse model, sera from control and AH patients did not differ significantly in recognition of JHDN-1 (Fig. 1F); JHDN-6, which differed from JHDN-5 by only 3 bp; or any other epitope (Fig. S1A to D). Additionally, SYFPEITHI queries uncovered several human MHC II haplotypes with favorable binding coefficients for JHDN-5 (Table 2). Thus, our findings support JHDN-5 as a CYP2E1 epitope that may also be responsible for hepatitis or CYP2E1 autoantibodies in AH patients and suggest that this epitope could be the dominant CYP2E1 epitope in anesthetic hepatitis.

**TABLE 2:** SYFPEITHI prediction score for human MHC II haplotypes

Human MHC II haplotype	SYFPEITHI score
HLA-DRB1*0101	18
HLA-DRB1*0701	18
HLA-DRB1*0401	16
HLA-DRB1*1501	12
HLA-DRB1*1101	11
HLA-DRB1*1301	10

10.1128/mSphere.00453-18.1 FIG S1 Epitope analysis, verification, and hybridoma serum formulation. (A to D) Human serum (1:100) from patients with anesthetic hepatitis (AH, *n* = 44) and controls (*n* = 45) was tested for antibodies to epitopes (0.5 µg/100 µl) by ELISA using alkaline phosphatase-conjugated IgG (30 min) secondary antibodies. Optical density was measured at 405 nm. Sera from control and AH patients did not differ significantly in recognition of JHDN-2 (A), JHDN-4 (B), JHDN-6 (C), or JHDN-9 (D). (E) Percent covalent modification of JHDN-5 by TFA haptens measured at 344 nm was calculated by comparing the optical density ratios of modified and native JHDN-5. We confirmed 34.5% modification of JHDN-5 by the TFA hapten. (F) Seven IgG-producing B cell hybridomas (circled in red) produced high levels of antisera against the JHDN-5 epitope. The remaining 5 hybridomas were not developed further because they did not produce high levels of JHDN-5 antisera. Download FIG S1, TIF file, 0.3 MB. Copyright © 2018 McCarthy et al. 2018 McCarthy et al. This content is distributed under the terms of the Creative Commons Attribution 4.0 International license.

### Covalent modification of JHDN-5 and IL-4 is required for the development of hepatitis in BALB/c mice.

**FIG 2:**
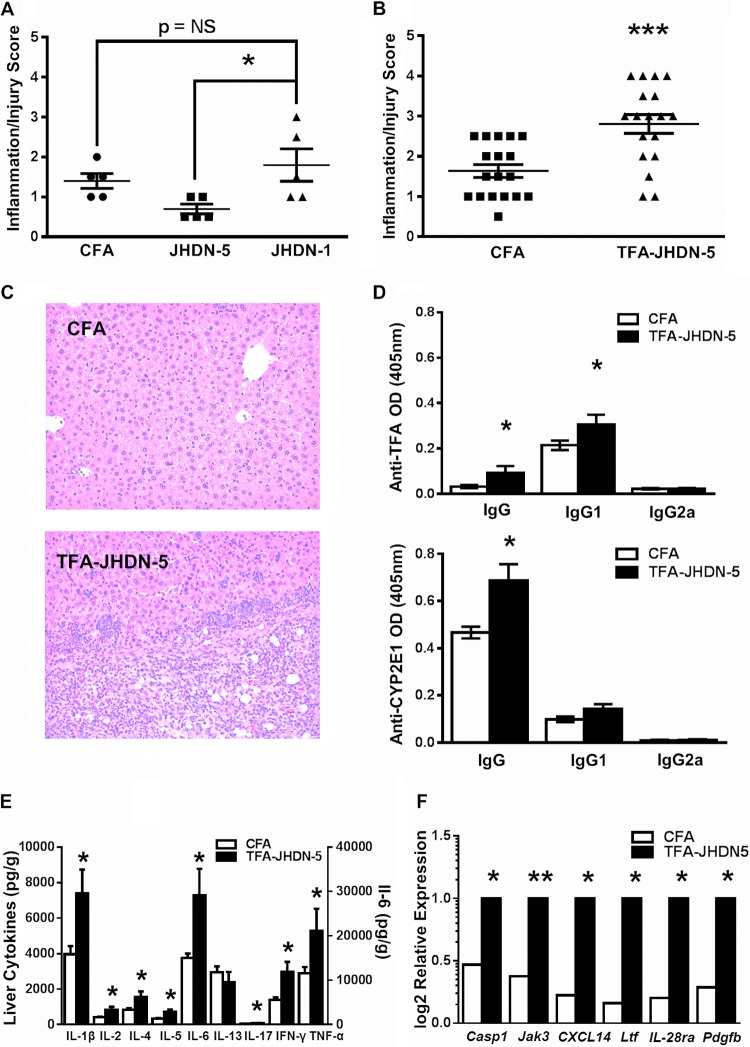
Covalent modification of JHDN-5 is critical for induction of hepatitis and antibodies in BALB/c mice. After 3 weeks, mice immunized on days 0 and 7 with CFA ± JHDN-5, JHDN-1, or TFA-JHDN-5 (100 µg) were evaluated for hepatitis and serum antibodies by ELISA (405 nm) using CYP2E1 test antigens (0.5 µg/100 µl), sera (1:100), and AKP-conjugated IgG, IgG1, and IgG2a secondary antibodies (1:1,000). (A) Inflammation/injury scores of liver sections from immunized BALB/c mice demonstrating that JHDN-1 (1.8 ± 0.9) induced more hepatitis than JHDN-5 (0.7 ± 0.3; *, *P* < 0.05) but not compared to mice immunized with CFA (1.4 ± 0.4) (mean ± SD). (B) Inflammation/injury scores of liver sections from immunized mice demonstrating that TFA-JHDN-5 induced more severe hepatitis (2.8 ± 0.9) than did CFA (1.6 ± 0.7); mean ± SD; ***, *P* < 0.001. (C) Representative liver sections (5 µm thick) stained with hematoxylin and eosin comparing CFA- and TFA-JHDN-5-immunized BALB/c mice, demonstrating increased hepatitis in the form of granulocytic (blue) infiltration in TFA-JHDN-5-immunized mice (64× magnification). (D) TFA-JHDN-5 increased TFA IgG, TFA IgG1, and CYP2E1 IgG more than did CFA; *, *P* < 0.05. (E and F) Hepatic levels of IL-1β, IL-2, IL-4, IL-5, IL-6, IL-17, IFN-γ, and TNF-α (E) as well as mRNA expression of *Casp1*, *Jak 3*, *CXCL14*, *IL-28ra*, and *Pdgfb* (F) were upregulated compared to CFA-immunized mice; *, *P* < 0.05; **, *P* < 0.01.

To test whether candidate epitopes induced drug-induced hepatitis and CYP2E1 antibodies, we immunized BALB/c mice with complete Freund’s adjuvant (CFA) ± JHDN-5 or JHDN-1 emulsified in CFA on days 0 and 7, as previously described (3). At 3 weeks, none of the epitopes induced CYP2E1 autoantibodies. JHDN-1 induced a low level of hepatitis that was not significantly greater than CFA alone and interestingly greater than JHDN-5 (Fig. 2A and Fig. S2A). The JHDN-5 epitope contains a centrally located lysine (K, Lys^123^) (Table S1B). We prepared a new immunogen by modifying Lys^123^ with the TFA hapten (25) and confirmed 34.5% modification of JHDN-5 by the TFA hapten (Fig. S1E). JHDN-1 was not altered because it does not contain lysine (Table S1A).

10.1128/mSphere.00453-18.2 FIG S2 Unaltered epitope induces low levels of hepatitis similar to CFA, and JHDN-5 antiserum colocalizes with CYP2E1, mitochondria, and Golgi. (A) Representative liver sections (5 µm thick) stained with hematoxylin and eosin comparing CFA alone and JHDN-5 and JHDN-1 emulsified in CFA-immunized BALB/c mice demonstrating increased hepatitis in the form of granulocytic (blue) infiltration in JHDN1-immunized mice compared to JHDN-5 but not mice immunized with CFA alone (64× magnification). (B) Representative confocal images of HepaRG cells stained with Alexa Fluor 488-labeled JHDN-5 antiserum (1:100) in addition to Alexa Fluor 594-labeled CYP2E1 (1:100) demonstrating colocalization of JHDN-5 antiserum and CYP2E1. (C) Pearson’s colocalization analysis demonstrating similar levels of colocalization when comparing Alexa Fluor 488-labeled JHDN-5 antiserum in combination with Alexa Fluor 594-labeled CYP2E1 IgG or Alexa Fluor 488-labeled JHDN-5 antiserum in combination with MitoTracker Red (1:100). (D) Representative confocal images of HepaRG cells stained with Alexa Fluor 488-labeled JHDN-5 antiserum (1:100) in addition to BODIPY Red (Golgi, 1:100), demonstrating minimal colocalization with Golgi. (E) Pearson’s colocalization analysis confirming that JHDN-5 IgG colocalized with Alexa Fluor 488-labeled JHDN-5 IgG with BODIPY Red (Golgi) similar to that of mouse IgG (control). Download FIG S2, TIF file, 0.8 MB. Copyright © 2018 McCarthy et al. 2018 McCarthy et al. This content is distributed under the terms of the Creative Commons Attribution 4.0 International license.

TFA-JHDN-5 immunizations induced significantly more hepatitis (Fig. 2B and C), serum levels of TFA antibodies, CYP2E1 autoantibodies (Fig. 2D), and hepatic tissue levels of interleukin (IL)-1β, IL-2, IL-4, IL-5, IL-6, IL-17, interferon (IFN)-γ, and tumor necrosis factor (TNF)-α by 3 weeks (Fig. 2E). This finding suggested that posttranslational modification of JHDN-5 was required for the development of hepatitis, TFA antibodies, and CYP2E1 autoantibodies. Histological sections revealed ASD esterase-positive cells, similarly identified in the original description of this model (3) (Fig. S3 and S4). We also found significant upregulation of caspase 1 (*Casp1*), a key inflammation mediator (26); Janus kinase 3 (*Jak 3*), a key controller of signal transduction after receptor activation by the common γ chain (27); and platelet-derived growth factor (*Pdgfb*), a key regulator of hepatic fibrosis (28). In addition, we detected significant upregulation of chemokine (C-X-C motif) ligand 14 (*CXCL14*), a potent chemoattractant for monocytes, dendritic cells, and NK cells seen in other forms of toxic liver injury (29), and upregulation of *IL-28ra*, which has been associated with improved outcomes in viral hepatitis when highly expressed on neutrophils (30) (Fig. 2F). These findings suggested that TFA-JHDN-5 could induce hepatitis, CYP2E1 autoantibodies, and profibrotic genes. However, fibrosis has not been associated with experimental drug-induced hepatitis in mice, suggesting that profibrotic signals may be downregulated by *IL-28ra* (30).

10.1128/mSphere.00453-18.3 FIG S3 JHDN-5 antiserum induces oxidative stress in HepaRG cells. Confocal images of HepaRG cells grown for 7 days on fibronectin-covered coverslips and then treated for 2 hours with (A) mouse IgG (1:100, negative control), (B) JHDN-5 antiserum (1:100), or (C) *tert*-butyl hydroperoxide (TBHP, 150 µM, positive control) followed by Cell Rox Deep Red. (D) Oxidative stress levels were higher after treatments with JHDN-5 IgG or TBHP than after treatment with mouse IgG; *, *P* < 0.05; ***, *P* < 0.001, respectively. JHDN-5-induced oxidative stress was not significantly different from that induced by TBHP. Download FIG S3, TIF file, 0.4 MB. Copyright © 2018 McCarthy et al. 2018 McCarthy et al. This content is distributed under the terms of the Creative Commons Attribution 4.0 International license.

10.1128/mSphere.00453-18.4 FIG S4 Naphthol-ASD chloroacetate esterase detection in BALB/c mouse liver sections three weeks following immunizations with CFA ± TFA-JHDN-5. BALB/c mice were immunized with CFA ± TFA-JHDN-5 (100 µg). Representative paraffin liver sections (5 µm thick) stained with naphthol-ASD chloroacetate esterase stain comparing CFA- and TFA-JHDN-5-immunized BALB/c mice demonstrating increased granulocytic (blue) infiltration (black arrows) in TFA-JHDN-5-immunized (A) compared to CFA-immunized (B) mice (64× magnification). Download FIG S4, TIF file, 1.5 MB. Copyright © 2018 McCarthy et al. 2018 McCarthy et al. This content is distributed under the terms of the Creative Commons Attribution 4.0 International license.

**FIG 3:**
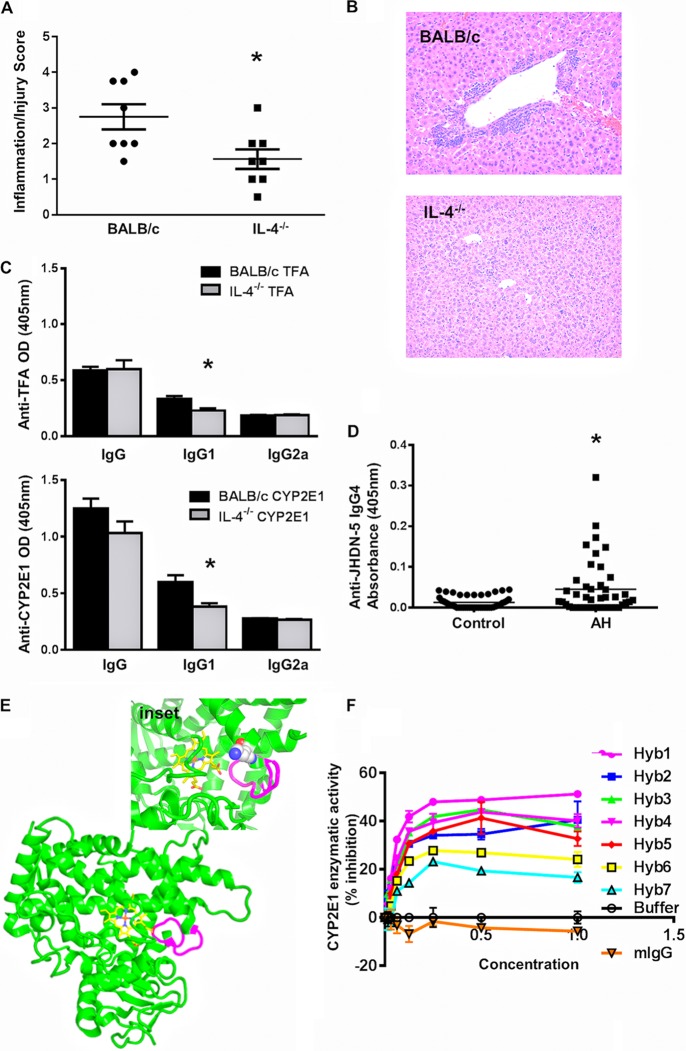
IL-4 promotes TFA-JHDN-5-induced responses. BALB/c and IL-4^−/−^ mice were immunized with TFA-JHDN-5 and evaluated after 3 weeks. (A) Inflammation/injury scores of liver sections from immunized BALB/c and IL-4^−/−^ mice demonstrating that TFA-JHDN-5 induced more severe hepatitis in BALB/c (2.8 ± 0.9) than IL-4^−/−^ (1.6 ± 0.8) mice; mean ± SD; *, *P* < 0.05. (B) Representative liver sections (5 µm thick) stained with hematoxylin and eosin comparing TFA-JHDN-5-immunized BALB/c and IL-4^−/−^ mice demonstrating increased hepatitis in the form of granulocytic (blue) infiltration in TFA-JHDN-5-immunized BALB/c mice compared to IL-4^−/−^ mice (64× magnification). (C) TFA and CYP2E1 IgG1 levels were lower in IL-4^−/−^ than BALB/c mice by ELISA (405 nm) using mouse sera (1:100) and AKP-conjugated IgG, IgG1, and IgG2a secondary antibodies (1:1,000); *, *P* < 0.05. (D) AH patients (*n* = 44, 1:100) demonstrated higher serum levels of JHDN-5 IgG4 than controls (*n* = 45; *, *P* < 0.05) by ELISA (405 nm) using AKP-IgG4 secondary antibodies (1:1,000). (E) Placement of an epitope antibody that would recognize JHDN-5 (magenta). Theoretically, this antibody would block access to the central heme molecule (carbon, yellow; oxygen, red; iron, red; nitrogen, blue). Inset: proposed modification of Lys^123^ in the JHDN-5 epitope within the CYP2E1 active site by the trifluoroacetyl chloride hapten. Lys123 is described in the linear epitope and identified by the TFA molecules that are attached to it (red, white, and blue circles). (F) Seven B cell hybridomas raised against the JHDN-5 epitope produced antisera that diminished CYP2E1 activity in human microsomes up to 50%.

IL-4^−/−^ mice immunized with TFA-JHDN-5 developed significantly less hepatitis and TFA and CYP2E1 autoantibodies than did BALB/c mice (Fig. 3A to C), suggesting that IL-4 promotes TFA-JHDN-5-induced hepatitis and antibodies, a finding similar to that in our prior studies (9). To uncover roles for JHDN-5 and IL-4 in AH, we tested their sera for IL-4-associated, immunoglobulin subclass 4 (IgG4) antibodies because this subclass previously had been found to be IL-4 responsive (31) and also had been detected in AH (12). We found significantly higher levels of JHDN-5 IgG4 autoantibodies in AH than in control patients (Fig. 3D), strengthening our notion that IL-4 and JHDN-5 are strongly associated with anesthetic hepatitis and CYP2E1 antibodies in mice and patients.

### JHDN-5 antiserum blocks CYP2E1 activity *in vitro*.

Autoantibodies to JHDN-5 were rarely detected in BALB/c or IL-4^−/−^ mice; thus, we could not address whether JHDN-5 autoantibodies had pathogenic associations in mice. To test this possibility, we raised antisera against the JHDN-5 epitope using seven IgG-producing B cell hybridomas that recognize JHDN-5 in enzyme-linked immunosorbent assays (ELISAs) (Fig. S1F). Using CYP2E1-expressing microsomes, we found that antisera from all 7 hybridomas inhibited CYP2E1 activity up to 50% *in vitro* (Fig. 3F), suggesting functional consequences for JHDN-5 IgG. To explain our findings, we mapped the location of JHDN-5 and the potential binding site of a JHDN-5-specific antibody using the suggested 3-dimensional structure of CYP2E1 (32). Structurally, JHDN-5 IgG could block the entrance of drugs metabolized by CYP2E1 in its present conformation (Fig. 3E) and possibly block CYP2E1 enzyme activity. We also determined that the single lysine in the JHDN-5 epitope was within the active site and accessible for TFA modification (Fig. 3E, inset).

### JHDN-5 IgG colocalizes with mitochondria and ER in HepaRG cells.

**FIG 4:**
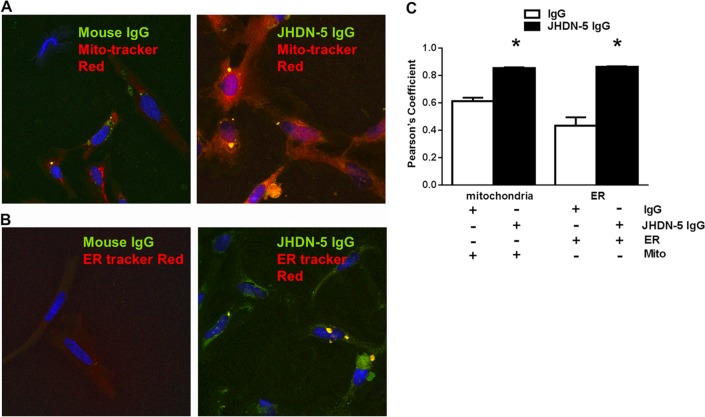
JHDN-5 antiserum recognizes mitochondria and endoplasmic reticulum (ER) in HepaRG cells. (A and B) Confocal images of HepaRG cells stained with Alexa Fluor 488-labeled JHDN-5 or mouse IgG (1:100) in addition to Alexa Fluor 594-labeled MitoTracker Red (1:100) or ER-Tracker Red (1:100). (C) Alexa Fluor 488-labeled JHDN-5 IgG colocalized with MitoTracker Red and ER-Tracker Red to a greater degree than with Alexa Fluor 488-labeled mouse IgG control; *, *P* < 0.05.

To determine if JHDN-5 IgG recognized CYP2E1 in cell cultures, we utilized HepaRG cells, a human progenitor cell line capable of differentiating into biliary and hepatocyte-like cells with functional properties similar to adult hepatocytes (33). Alexa Fluor 488-labeled JHDN-5 IgG colocalized with MitoTracker Red and ER-Tracker Red significantly more than Alexa Fluor 488-labeled mouse IgG (control) (Fig. 4A to C). In fact, JHDN-5 IgG colocalized with Alexa Fluor 594-labeled CYP2E1 IgG at levels similar to MitoTracker Red (Fig. S2A and B), suggesting that JHDN-5 IgG most likely recognized CYP2E1 in mitochondria and possibly the endoplasmic reticulum (ER), raising the possibility that this antiserum could modulate CYP2E1 activity in intact cells. Interestingly, colocalization of Alexa Fluor 488-labeled JHDN-5 IgG with BODIPY Red (Golgi) was similar to that of mouse IgG (control) (Fig. S2C and D).

### JHDN-5 IgG upregulates mitochondrial oxidative stress and heat shock protein 27 in HepaRG cells.

**FIG 5:**
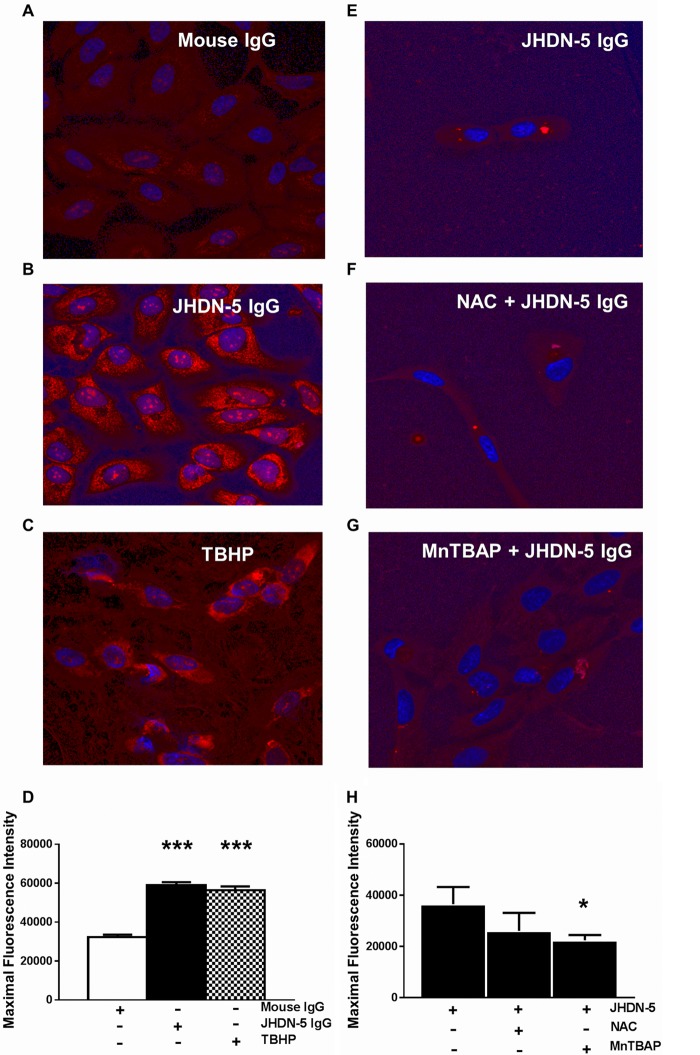
JHDN-5 antiserum induces mitochondrial oxidative stress in HepaRG cells. (A to C) Confocal images of HepaRG cells treated for 2 h with (A) mouse IgG (1:100, negative control), (B) JHDN-5 antiserum (1:100), or (C) TBHP (150 µM, positive control) followed by MitoSOX Red. (D) Mitochondrial oxidative stress levels were higher after treatments with JHDN-5 IgG or TBHP than after treatment with mouse IgG; ***, *P* < 0.001. JHDN-5-induced mitochondrial oxidative stress was not significantly different from that induced by TBHP. Separate wells were treated with (E) JHDN-5 IgG alone, pretreated with (F) NAC, 200 µM, 1 h before JHDN-5 IgG, or (G) treated with MnTBAP, 100 µM, for the last 30 min of antibody treatment followed by MitoSOX Red reagent. (H) JHDN-5 IgG-induced oxidative stress was significantly lowered by MnTBAP; *, *P* < 0.05.

Demonstrating that JHDN-5 IgG recognized CYP2E1 in cells and decreased CYP2E1 enzymatic activity in human microsomes raised the possibility that JHDN-5 could disrupt critical processes such as mitochondrial respiration in intact cells, probably through production of reactive oxygen species (ROS). We found that JHDN-5 IgG increased generalized oxidative stress (*P* < 0.05, Fig. S3A to D), as indicated by CellRox Deep Red, as well as ROS through mitochondrial complex 1-specific inhibition, as indicated by MitoSOXRed superoxide indicator (*P* < 0.001, Fig. 5A and B) in HepaRG cells *in vitro*, compared with cells treated with mouse IgG. JHDN-5 IgG-induced mitochondrial oxidative stress was similar to that induced by the organic peroxide and positive-control *tert*-butyl hydroperoxide (TBHP) (Fig. 5B to D). Pretreatment with *N*-acetyl cysteine (NAC) did not reverse oxidative stress in HepaRG cells (Fig. 5F and H). In contrast, oxidative stress was significantly reversed by the mitochondrial superoxide dismutase mimetic Mn(III)tetrakis (4-benzoic acid)porphyrin chloride (MnTBAP; *P* < 0.05, Fig. 5G and H), suggesting that JHDN-5 IgG likely induced ROS through mitochondrial complex 1 inhibition in HepaRG cells.

**FIG 6:**
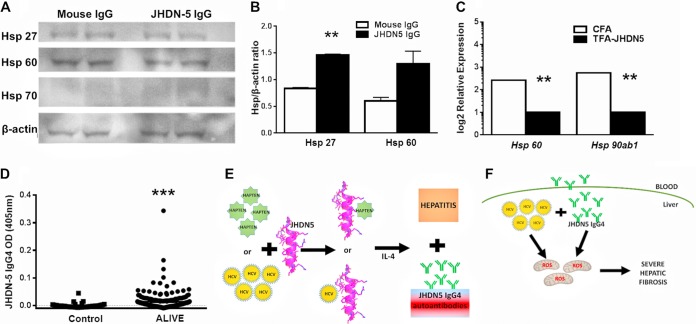
JHDN-5 antiserum upregulates HSP27 in HepaRG cells and fibrosis in viral hepatitis. (A and B) Band intensities of HSP were normalized and reported as HSP/β-actin ratios. JHDN-5 IgG upregulated HSP27 but not HSP60 in HepaRG cells more than did mouse IgG; **, *P* < 0.01. (C) TFA-JHDN-5 downregulated liver mRNA expression of HSP60 and HSP90 compared to CFA-immunized mice; *P* < 0.01 versus CFA. (D) JHDN-5 IgG4 was measured in serum samples (1:100) from patients in the ALIVE study group (*n* = 200) and from controls (*n* = 45) by ELISA as described for Fig. 3D. JHDN-5 IgG4 levels were higher in ALIVE patients than in controls; ***, *P* < 0.001. (E) We propose that posttranslational modification of JHDN-5 induces hepatitis and JHDN-5 IgG4 in patients with anesthetic and viral hepatitis. (F) In susceptible persons with viral hepatitis (possibly HCV), JHDN-5 IgG4 synergistically upregulates hepatic fibrosis via mitochondrial oxidative stress.

To uncover additional sequelae from JHDN-5-mitochondrion interactions, we investigated heat shock protein (HSP) expression in HepaRG cells treated with mouse or JHDN-5 IgG as well as mRNA expression in livers from TFA-JHDN-5-immunized mice. We found that HSP27 but not HSP60 protein levels were significantly higher in HepaRG cells treated with JHDN-5 IgG than in those treated with mouse IgG (Fig. 6A and B), HSP60 and HSP90 mRNA levels were significantly downregulated in TFA-JHDN-5-immunized mice (Fig. 6C). We did not detect HSP70 protein or mRNA levels. Prior investigators have demonstrated that HSP27 blocks intracellular ROS attack on mitochondria (34) while HSP60, 70, and 90 promote cellular survival (35). Thus, our findings suggest that JHDN-5 antiserum triggers mitochondrial oxidative stress via complex 1 inhibition and ROS, which subsequently upregulates HSP27. Additionally, TFA-JHDN-5 negatively impacts hepatic cellular survival.

### Sera from patients with viral hepatitis recognize JHDN-5.

Because antisera from the CYP2E1 epitope JHDN-5 induced ROS and mitochondrial oxidative stress *in vitro*, we hypothesized that JHDN-5 antibodies may positively correlate with hepatic fibrosis in viral hepatitis. Mitochondrial oxidative stress may promote inflammation or hepatic fibrosis. Hepatitis C virus (HCV) core proteins can promote mitochondrial oxidative stress (21, 22); however, roles for CYP2E1-induced oxidative stress have not been completely clarified. CYP2E1 IgG has been detected in patients with chronic hepatitis C (CHC) (14, 15).

We measured JHDN-5 IgG4 in sera from a random sample of patients from the ALIVE (AIDS- Linked to Intravenous Experience) study (36). The majority of patients (*n *=* *200) in our sample were African American, HCV antibody positive, and human immunodeficiency virus (HIV) negative, and demonstrated a low or moderate liver elastography score (Table 3). We detected higher levels of JHDN-5 IgG4 in the ALIVE sera than in control samples (*P* < 0.001, Fig. 6D). Higher levels of JHDN-5 IgG4 were associated with moderate (*P* = 0.0106) or severe (*P* = 0.0126) degrees of liver fibrosis as measured by liver elastography (Table 4). After controlling for significant covariates, we found that higher levels of JHDN-5 IgG4 were associated with severe hepatic fibrosis in our random sampling of patients with viral hepatitis (*P* = 0.0142, Table 5). Taken together with our *in vitro* studies, this may suggest that JHDN-5 IgG4-induced mitochondrial oxidative stress contributes to the severity of fibrosis, possibly through inhibition of mitochondrial complex 1 and induction of ROS. However, there are many autoantibodies seen in viral hepatitis. Linear regression analysis showed that higher levels of JHDN-5 IgG4 autoantibody correlations with HCV antibody positivity approached but did not reach statistical significance (*P* = 0.0614). In addition, further examination of HCV antibody-positive persons showed that higher levels of JHDN-5 IgG4 autoantibody correlations with detectable viral loads almost reached statistical significance in our random sample set (*P* = 0.0506) (Table 4). Hence, we demonstrate a shared CYP2E1 epitope between anesthetic and viral hepatitis.

**TABLE 3:** Demographic data for serum samples from the ALIVE study

Characteristic^*a*^	Frequency or value
Age, mean ± SD	52 ± 7.9
Male, *n* (%)	118 (59.6)
Female, *n* (%)	80 (40.4)
Non-African American, *n* (%)	19 (9.6)
African American, *n* (%)	179 (90.4)
HIV antibody, *n* (%)	
Negative	122 (62)
Positive	76 (38)
HCV, *n* (%)	
Antibody	
Negative	26 (13.1)
Positive	172 (86.9)
Viral load	
Nondetectable	28 (16.7)
Detectable	114 (67.9)
Elastography result (kPa)	
Mean ± SD	10.1 ± 11.4
<8, *n* (%)	122 (68.5)
8–12.3, *n* (%)	26 (14.6)
≥12.3, *n* (%)	30 (16.9)
JHDN-5 IgG4 optical density, mean ± SD	0.014 ± 0.032

a Abbreviations: ALIVE, AIDS-Linked to Intravenous Experience; HCV, hepatitis C virus; HIV, human immunodeficiency virus; SD, standard deviation.

**TABLE 4:** Linear regression analysis of log_10_-transformed JHDN-5 IgG4 optical density

Covariate^*a*^	Estimate	95% confidence limit	*P* value^*b*^
Female	−0.23	−0.41 to −0.04	**0.0170**
African American	−0.25	−0.56 to 0.06	0.1150
Age per 10 yr	0.04	−0.07 to 0.16	0.4644
HIV positive	−0.08	−0.27 to 0.11	0.4247
HBV surface Ag	0.43	−0.22 to 1.07	0.1986
Liver elastography (kPa)			
Linear	0.01	0.00 to 0.02	**0.0106**
<8	Reference value		
8–12.2	0.06	−0.21 to 0.34	0.6556
≥12.3	0.33	0.07 to 0.59	**0.0126**
HCV			
Ab negative	Reference value		
Ab positive	0.26	−0.01 to 0.53	0.0614
VL not detectable	0.23	−0.16 to 0.59	0.1879
VL detectable	0.28	−0.00 to 0.56	0.0506

a Abbreviations: Ab, antibody; Ag, antigen; HBV, hepatitis B virus; HCV, hepatitis C virus; HIV, human immunodeficiency virus; VL, viral load.

b Bold indicates significance.

**TABLE 5:** Multivariate analysis linear regression model of log_10_-transformed JHDN-5 IgG4

Covariate	Reference value	95% confidence limits	*P* value^*a*^
Female	−0.18	−0.38 to 0.02	0.0793
Age per 10 yr	0.05	−0.08 to 0.18	0.4463
African American	−0.33	−0.66 to 0.00	0.0532
Liver elastography (kPa)			
<8	1.00		
8–12.2	0.08	−0.20 to 0.36	0.5539
≥12.3	0.33	0.07 to 0.59	**0.0142**

a Bold indicates significance.

## DISCUSSION

We describe a connection between anesthetic and viral hepatitis wherein a cytochrome p4502E1 (CYP2E1) epitope, glycine^113^-leucine^135^ (Gly^113^-Leu^135^, JHDN-5), generates autoantibodies that are detectable in the sera of patients in both diseases. In mice, JHDN-5 initiates anesthetic hepatitis and CYP2E1 autoantibodies in an interleukin-4 (IL-4)-dependent manner that requires covalent modification of lysine^123^ (Lys^123^) by a trifluoroacetyl chloride (TFA) drug metabolite. In addition, TFA-JHDN-5 upregulates hepatic proinflammatory and profibrotic mRNA while cellular survival signals are downregulated in mice. IL-4-associated JHDN-5 IgG4 autoantibodies are detectable in patients with anesthetic and viral hepatitis. Significant correlation of elevated JHDN-5 IgG4 levels with severe hepatic fibrosis in viral hepatitis patients may suggest additional associations for autoantibodies with fibrosis. We have first uncovered functional roles for JHDN-5 IgG that include inhibition of CYP2E1 enzyme activity, induction of mitochondrial oxidative stress via complex 1 inhibition, and upregulation of reactive oxygen species (ROS)-sensitive heat shock protein 27 (HSP27) in liver cell cultures. Taken together, our report identifies one shared epitope between anesthetic and viral hepatitis that may contribute to sequelae following exposure to drugs or viruses and strongly suggests that this epitope could be the dominant CYP2E1 epitope in anesthetic hepatitis.

We found that JHDN-5 is recognized by mouse splenocytes in experimental drug-induced hepatitis (3). Our evidence suggests that JHDN-5 is most likely recognized by major histocompatibility 2 (MHC II) cells bearing I-Ed whose positively charged core (Table 1B) may promote recognition by the I-Ed haplotype (37) and limit its recognition by I-Ad (38). SYFPEITHI prediction confirms favorable binding for JHDN-5 and I-Ed while clarifying unfavorable binding for I-Ad (Table 1). Even so, differences in binding could have been affected by our choice to use the human version of the epitope sequence. Human CYP2E1 has 78% sequence homology with the mouse and the JHDN-5 epitope shares 85% sequence homology with the same location of mouse CYP2E1. Isoleucine^125^ in human CYP2E1 is valine^125^ in mouse CYP2E1; however, both are nonpolar with uncharged, aliphatic R groups. Threonine^131^ in human CYP2E1 is serine^131^ in mouse CYP2E1, and both are polar with uncharged R groups with minimal differences where a methyl group in the human form is replaced by a hydrogen in the mouse form. Threonine^132^ in human CYP2E1 is isoleucine^132^ in mouse CYP2E1. In this difference, a polar amino acid is exchanged for a nonpolar amino acid; however, both have uncharged R groups. Interestingly, the Ser^129^ position was conserved in mouse and human CYP2E1. Thus, amino acid differences between human and mouse JHDN-5 should not affect our observed immune responses.

We also showed that sera from anesthetic hepatitis and ALIVE (AIDS-Linked to Intravenous Experience) patients recognize JHDN-5. Moreover, SYFPEITHI-identified human MHC II haplotypes that recognize JHDN-5 have been associated with liver disease, where human leukocyte antigens (HLA) DRB1 1101 and 0401 have been associated with hepatitis C virus (HCV) clearance (39, 40) and HLA DRB1 0701 has been associated with autoimmune hepatitis type 2, another cytochrome p450-triggered hepatitis (41). HLA DRB1 0701 has also been associated with protection from drug-induced liver injury associated with amoxicillin-clavulanate, and HLA DRB1 0501 has been associated with a 10-fold-increased risk for developing drug-induced liver injury from the same drug (42).

We show that posttranslational modification of Lys^123^ in JHDN-5 is required to induce hepatitis and CYP2E1 autoantibodies in BALB/c mice, confirming earlier hypotheses described in halothane toxicity (11). TFA-JHDN-5 also upregulates caspase 1, resulting in proinflammatory cytokines as well as common γ chain cytokines IL-2 and IL-4 via Jak3. IL-4 has been associated with the initiation of drug-induced hepatitis. Our studies also suggest that immune cells are recruited to the liver via CXCL14 (chemokine [C-X-C motif] ligand 14). IL-28ra may downregulate this process in mice similar to other forms of hepatitis.

JHDN-5 IgG inhibits CYP2E1 activity up to 50% but not 100%, which may reflect antiserum concentration or different binding affinities by the antiserum when exposed to microsomes *in vitro*. Enzyme inhibition introduces the possibility of a functional autoantibody. CYP2E1 antibodies have been pathogenically associated with chronic hepatitis C (CHC) (15). Reduced CYP2E1 enzyme activity has been reported in alcoholic hepatitis, and these patients can express CYP2E1 autoantibodies (43); however, in alcoholic hepatitis, reduced CYP2E1 activity has been associated with increased severity of hepatitis and not the antibody itself.

We first show that JHDN-5 IgG can undergo transmembrane migration into HepaRG cells and preferentially target mitochondria and endoplasmic reticula, most likely because of their CYP2E1 content. Mitochondrial oxidative stress is most likely triggered by superoxide anions formed following JHDN-5 IgG-mitochondrial CYP2E1 interactions since it was detected by MitoSOX, a fluorescent antibody that specifically targets superoxide anions following complex 1 inhibition. More importantly, oxidative stress was reversed by the mitochondrial matrix mimetic, MnTBAP (44), but not the generic antioxidant NAC. Mitochondrial oxidative stress was further implicated by upregulation of HSP27 that targets ROS. Thus, our studies suggest a direct pathogenic effect for JHDN-5 autoantibodies via complex 1 inhibition and mitochondrial oxidative stress, which contradicts currently held beliefs that these autoantibodies are epiphenomena. Mitochondrial oxidative stress could then potentially contribute to either the initiation or progression of liver injury. We also show that JHDN-5 and mouse IgG similarly colocalize with the Golgi apparatus. A prior study detected enzymatically active CYP2E1 in the Golgi (45). We are currently investigating whether JHDN-5 IgG modulates Golgi functions or other metabolic functions *in vivo*.

We show that female patients with viral hepatitis have significantly lower levels of JHDN-5 IgG4 than do males. We have previously shown that women develop higher levels of CYP2E1 IgG autoantibodies than men after anesthetic exposure (46). What separates the current studies from our prior work is that we have directly associated JHDN-5 IgG4 with oxidative stress and severe hepatic fibrosis. Another explanation for the finding is that the association of JHDN-5 IgG4 with greater liver fibrosis might instead reflect higher immunoglobulin levels in general, which have been reported with cirrhosis.

Oxidative stress may accelerate the progression to hepatic fibrosis in patients with CHC (47). Oxidative stress may promote activation of hepatic stellate cells (48). Interestingly, immunization of BALB/c mice with TFA-JHDN-5 upregulated *Pdgfb* mRNA, a potent proproliferative cytokine for hepatic stellate cells (28), supporting a connection between JHDN-5 and fibrosis. However, JHDN-5 IgG4 was detected in patients with viral and anesthetic hepatitis, while hepatic fibrosis is not associated with anesthetic hepatitis, and we did not find histological evidence for increased collagen deposition in the BALB/c mice immunized with TFA-JHDN-5 (Fig. 2C). Thus, JHDN-5 IgG4-induced oxidative stress may provide an additional profibrotic response that increases the severity of hepatic fibrosis in viral hepatitis (Fig. 6D) but may not induce fibrosis by itself.

In our studies, we did not test for antibody-dependent, cell-mediated cytotoxicity because we were able to show direct antibody-induced effects on hepatocytes. Antibody-dependent, cell-mediated cytotoxicity has been demonstrated in tienilic acid-induced hepatitis (49). Hence, investigating the role of antibody-dependent, cell-mediated cytotoxicity in anesthetic drug-induced hepatitis is a subject of future studies in our lab.

This is the first demonstration of a common, MHC-restricted CYP2E1 epitope in patients with anesthetic hepatitis and with hepatic fibrosis. We show evidence that strongly suggests that this epitope could be the dominant CYP2E1 epitope in anesthetic hepatitis. We propose that posttranslational modification of JHDN-5 induces hepatitis and JHDN-5 IgG4 (Fig. 6C) and that these JHDN-5 autoantibodies inhibit CYP2E1 enzymatic activity. In susceptible persons, possibly those with viral hepatitis, JHDN-5 IgG4 synergistically promotes hepatic fibrosis via mitochondrial oxidative stress (Fig. 6D), which suggests a role for autoantibodies and possibly B cells in this disease (50). Although it is possible that our findings in viral hepatitis might be skewed by higher immunoglobulin levels in general, which have been reported with cirrhosis, our findings may elucidate one mechanism that contributes to the understanding of sequelae seen in liver injury from drugs or viruses. Additionally, in conjunction with prior studies identifying conformational CYP2E1 epitopes (16), our identification of MHC-restricted CYP2E1 epitopes can be utilized to develop specific diagnostic tests for drug-induced or viral hepatitis or associated fibrosis or to predict individuals at risk for developing these diseases or their sequelae.

## MATERIALS AND METHODS

### Sample size. (i) Mouse.

From our study (3), a sample size of 16 in each group would detect a histologic difference of 0.31 with sufficient power (0.8) and a significance level alpha (α) of 0.05 (two-tailed) (GraphPad StatMate 2.0). Experiments were repeated 4 times with 4 to 5 mice/group. Statistical outliers were detected using Grubbs’ test.

### (ii) Human.

In a prior study, CYP2E1 autoantibodies were increased in CHC compared to controls (14); the difference by ELISA was 0.150 ± 0.250 (mean ± SD, optical density [OD]). Forty-five samples would give sufficient power of 0.8 with α = 0.05 (Sigma Stat version 3.1).

### Mice.

Age-matched, 8- to 10-week-old female BALB/c and female IL-4-deficient (IL-4^−/−^) mice on a BALB/c background (Jackson Laboratory, Bar Harbor, ME) were maintained under pathogen-free conditions. All procedures were approved by the Johns Hopkins University Animal Care and Use Committee.

### Human CYP2E1 epitopes.

Human and mouse CYP2E1 sequences (Swiss-Prot) were entered into RANKPEP prediction of binding peptides, imed.med.ucm.es/Tools/rankpep.html. Common candidate epitopes were evaluated between mouse haplotypes I-Ad and I-Ed.

### DO11.10 T cell competitive inhibition assay.

DO11.10 T cell hybridomas were cultured with 0.5 mg OVA^323–329^ in complete medium, with increasing concentrations of each of the candidate epitopes. The supernatant was removed and cultured with CTLL-2 cells (ATCC, Manassas, VA). that would respond to IL-2. Proliferation of CTLL-2 cells measured by [^3^H]thymidine incorporation confirmed IL-2 production.

### Proliferation assays.

Mice were immunized with liver proteins covalently altered by a TFA drug hapten (TFA-S100) as previously described (3). Two weeks later, splenocytes were challenged with medium ± candidate CYP2E1 epitopes (10 µg/ml) or concanavalin A (ConA) (5 µg/ml, positive control). Proliferation was measured after 48 h by [^3^H]thymidine incorporation (51).

### SYFPEITHI epitope prediction.

MHC class II ligands were 15-mer with N- and C-terminal flanking residues. The nonamer core was within the MHC II binding groove. Prediction was based on published motifs. To calculate a score, ideal anchors are given 10 points, unusual anchors 6 to 8 points, auxiliary anchors 4 to 6 points, and preferred residues 1 to 4 points. Amino acids having a negative effect on binding ability are assigned values between −1 and −3. Since scores can vary, OVA^323–339^ and HEL^107–116^, known peptides recognized by I-Ad and I-Ed, respectively, were queried for comparison (52).

### Sera. (i) Hybridoma.

Hybridoma sera were made using the ClonaCell-HY hybridoma kit from Stem Cell Technologies by following the kit instructions. Briefly, after collecting a baseline serum sample prior to immunizations, BALB/c mice were immunized subcutaneously, four times with JHDN-5 (50 µg) emulsified in complete Freund’s adjuvant (CFA) at 7-day intervals. Two weeks following each immunization, a small sample of blood was obtained by intraperitoneal sampling, assessed for antibodies by ELISA, and compared to the baseline sample. All mice had good titers of antibodies. As recommended, 4 days before the day of fusion, mice were boosted with 50 µg of antigen in saline without adjuvant in a maximum volume of 200 µl. BALB/c mouse spleens were isolated, and single cell suspensions were formulated. Approximately 2 × 10^7^ parental myeloma cells and 1 × 10^8^ viable splenocytes were then fused with supplied myeloma cells using polyethylene glycol-mediated fusion, as per kit instructions. Hybridomas were selected using liquid medium using hypoxanthine-aminopterin-thymidine (HAT) medium. Supernatants were screened for antibody production by ELISA for responses to the CYP2E1 epitope and to the whole CYP2E1. Selected hybridomas were expanded, and the supernatants were reexamined by ELISA. Hybridoma serum was further purified (Proteus Protein G antibody purification kit, AbD Serotec) and tested for IgG to CYP2E1 or JHDN-5 (ELISA).

### (ii) Human (exempted by our IRB).

Anesthetic hepatitis samples were previously characterized (12) (see the supplemental material). The ALIVE (AIDS-Linked to Intravenous Experience) study is a prospective observational cohort study that was originally designed to characterize the incidence and natural history of HIV infection among injection drug users in Baltimore, MD (36). The study aims evolved over time to encompass access to care and impact of coinfections (e.g., HCV) and other related issues. Samples in this study were previously characterized by age, sex, race, HIV antibody status, HCV antibody and viral load status, HBV surface antigen status, and liver elastography, which was used to detect fibrosis by measuring liver stiffness by ultrasound in kilopascals (kPa). A normal liver elastography score was defined as <8 kPa. Mild to moderate fibrosis was defined as an elastography score of 8 to 12 kPa, and severe fibrosis was defined as a liver elastography score of >12.3 kPa.

### ELISA.

Assays were run (triplicates, 405 nm) using CYP2E1 and candidate epitope test antigens as well as TFA (0.5 µg/100 µl). Human serum (1:100) was assessed with alkaline phosphatase (AKP)-conjugated IgG (30 min) (Millipore, Billerica, MA) and IgG4 (90 min) secondary antibodies (Southern Biotech, Birmingham, AL) (9). Mouse and hybridoma sera (1:100) were assessed using AKP-conjugated IgG (Millipore), IgG1, and IgG2a (BD Biosciences, 30 min) (9).

### Hepatitis.

Mice were immunized with 100 µg of JHDN-1, JHDN-5, or JHDN-5+TFA covalent modification emulsified in CFA on days 0 and 7, in addition to 50 ng of pertussis toxin on day 0 (3), and euthanized 2 or 3 weeks after the initial immunizations. Control mice were similarly immunized with CFA alone.

### Cytokine and gene expression.

Livers were homogenized in 10% (wt/vol) RPMI-2% FCS (3), and cytokines were measured by ELISA (R&D Systems, Minneapolis, MN) and standardized per gram of tissue. Liver mRNA was analyzed (mouse inflammation panel, TaqMan qPCR array; Thermo Fisher)

### CYP2E1 activity.

Percent inhibition of CYP2E1 activity in microsomes (triplicates) treated *in vitro* with JHDN-5 IgG or normal mouse IgG (control, Santa Cruz Biotechnology, Dallas, TX) was measured using the Vivid CYP2E1 Blue screening platform (Invitrogen, Grand Island, NY).

### Histology. (i) Paraffin.

Liver tissue sections (0.5 µm thick) fixed in 10% neutral buffered formalin and stained with hematoxylin and eosin were scored for inflammation/injury: grade 0, no inflammation or necrosis; grade 1, minor periportal or lobular inflammation without necrosis; grade 2, periportal or lobular inflammation involving <50% of the section; grade 3, periportal or lobular inflammation involving ≥ 50% of the section or inflammation with necrosis; grade 4, inflammation with bridging necrosis (3).

### (ii) Fluorescence.

HepaRG cells were sparsely cultured (∼30% confluence) on fibronectin-covered coverslips for 7 days in dye-free Williams’s medium E supplemented as recommended. Alexa Fluor 488-conjugated mouse IgG or JHDN-5 (1:100) and Alexa Fluor 594-conjugated Mito-tracker Red or ER-tracker Red (1:100, ThermoFisher) were added for 2 h (37°C), mounted with ProLong Gold antifade reagent with DAPI (Cell Signaling, Danvers, MA), and examined by confocal microscopy (LSM700). Colocalization with mitochondria and ER was analyzed (Imaris).

### (iii) Oxidative stress detection.

HepaRG cells were incubated for 2 h in medium ± JHDN-5 or IgG (1:100). Additional wells received nothing (negative control) or TBHP (150 µM, positive control) for 1 h. Separate wells were either pretreated with NAC (200 µM) 1 h before antibody or TBHP treatment or treated with MnTbAP (100 µM) during the last 30 min. Maximal intensity of MitoSOX Red Superoxide indicator (ThermoFisher) was measured in 4 to 6 separate areas (ImageJ). The confocal images were obtained by a single blind image specialist where the control was based on the image with the lowest intensity.

### Western blotting.

Proteins from HepaRG cells treated with mouse or JHDN-5 IgG for 2 h were separated (25 µg/lane, 200 V) using 4 to 12% polyacrylamide ready-made minigels (Life Technologies) and transferred to nitrocellulose membranes (Iblot, Life Technologies). Membranes were probed with 1:1,000 HSP27 IgG (clone G31), HSP60 IgG (clone D6F1), or HSP70 IgG or β-actin IgG (clone 13E5, control) (Cell Signaling), followed by anti-mouse or anti-rabbit IgG HRP-linked secondary antibodies (1:5,000), and visualized with ECL (Amersham), and band intensities were analyzed using ImageJ software. Band intensities were normalized with β-actin and reported as HSP/β-actin ratios.

### Statistical analysis.

Experiments were analyzed with Mann-Whitney U (GraphPad Prism for Windows Version 6.04). Confocal studies were assessed using Pearson coefficient colocalization analysis and Mann-Whitney U. For ALIVE analyses, descriptive statistics examined the distribution of JHDN-5 IgG4. Because JHDN-5 IgG4 data had a skewed distribution, results were logarithmically transformed. Linear regression techniques analyzed the relationship between the outcome and covariates (SAS, version 9; SAS Institute, Cary, NC). A *P* value < 0.05 was considered significant.
