# Erratum for McCarthy et al., “Identification of a Shared Cytochrome p4502E1 Epitope Found in Anesthetic Drug-Induced and Viral Hepatitis”

**DOI:** 10.1128/mSphere.00654-18

**Published:** 2018-12-05

**Authors:** Elisa K. McCarthy, Amanda Vakos, Merylin Cottagiri, Joel J. Mantilla, Lakshmi Santhanam, David L. Thomas, L. Mario Amzel, Noel R. Rose, Dolores B. Njoku

**Affiliations:** a Department of Anesthesiology and Critical Care Medicine, Johns Hopkins University, Baltimore, Maryland, USA; b Division of Infectious Disease, Department of Medicine, Johns Hopkins University, Baltimore, Maryland, USA; c Department of Biophysics and Biophysical Chemistry, Johns Hopkins University, Baltimore, Maryland, USA; d Department of Pathology, Brigham and Women’s Hospital, Harvard University, Cambridge, Massachusetts, USA; e Department of Pathology, Johns Hopkins University, Baltimore, Maryland, USA

## ERRATUM

Volume 3, no. 5, e00453-18, 2018, https://doi.org/10.1128/mSphere.00453-18. This article was published on 10 October 2018 with the fourth author’s surname misspelled. The byline was updated in the current version, posted on 30 November 2018.

