# Supplementary material for: Identification of a Shared Cytochrome p4502E1 Epitope Found in Anesthetic Drug-Induced and Viral Hepatitis
Source: mSphere. 2018 Oct 10;3(5):e00453-18. doi: 10.1128/mSphere.00453-18 (PMC6180222; doi:10.1128/mSphere.00453-18)
Supplement: TABLE S1 [file sph005182659s1.pdf]

Supplemental Table 1A. Candidate epitopes generated with the Rankpep program

Epitope name		Sequence	
JHDN1	2	SAL/ GVT/ VAL/ LVW/ AAF/ LLL	19
JHDN2	75	QRM/ VVM/ HGY/ KAV/ KEA/ LLD	92
JHDN3	226	HYL/ PGS/ HRK/ VIK/ NVA/ EVK	243
JHDN4	377	IFR/ GYL/ IPK/ GTV/ WPT/ LDS	394

Four candidate epitopes of human CYP2E1 were selected with RANKpep as well as I-Ad and I-Ed mouse haplotypes (53). The underlined amino acids were added at the C terminus to create 18- to 20-mer peptides.

Supplemental Table 1B. Candidate epitopes centered on the CYP2E1 active site

Epitope Name		Sequence	
JHDN5	113	GII/ FNN/ GPT/ WKD/ IRR/ FSL/ TTL	133
JHDN6	116	FNN/ GPT/ WKD/ IRR/ FSL/ TTL/ RN	135
JHDN7	119	GPT/ WKD/ IRR/ FSL/ TTL/ RNY/ GM	138
JHDN8	122	WKD/ IRR/ FSL/ TTL/ RNY/ GMG/ K	140
JHDN9	125	IRR/ FSL/ TTL/ RNY/ GMG/ KQG/ NES	145

Five candidate epitopes were selected around the Serine^{129} CYP2E1 active site (53).
