# Supplementary figures and images for: Identification of a Shared Cytochrome p4502E1 Epitope Found in Anesthetic Drug-Induced and Viral Hepatitis
Source: mSphere. 2018 Oct 10;3(5):e00453-18. doi: 10.1128/mSphere.00453-18 (PMC6180222; doi:10.1128/mSphere.00453-18)

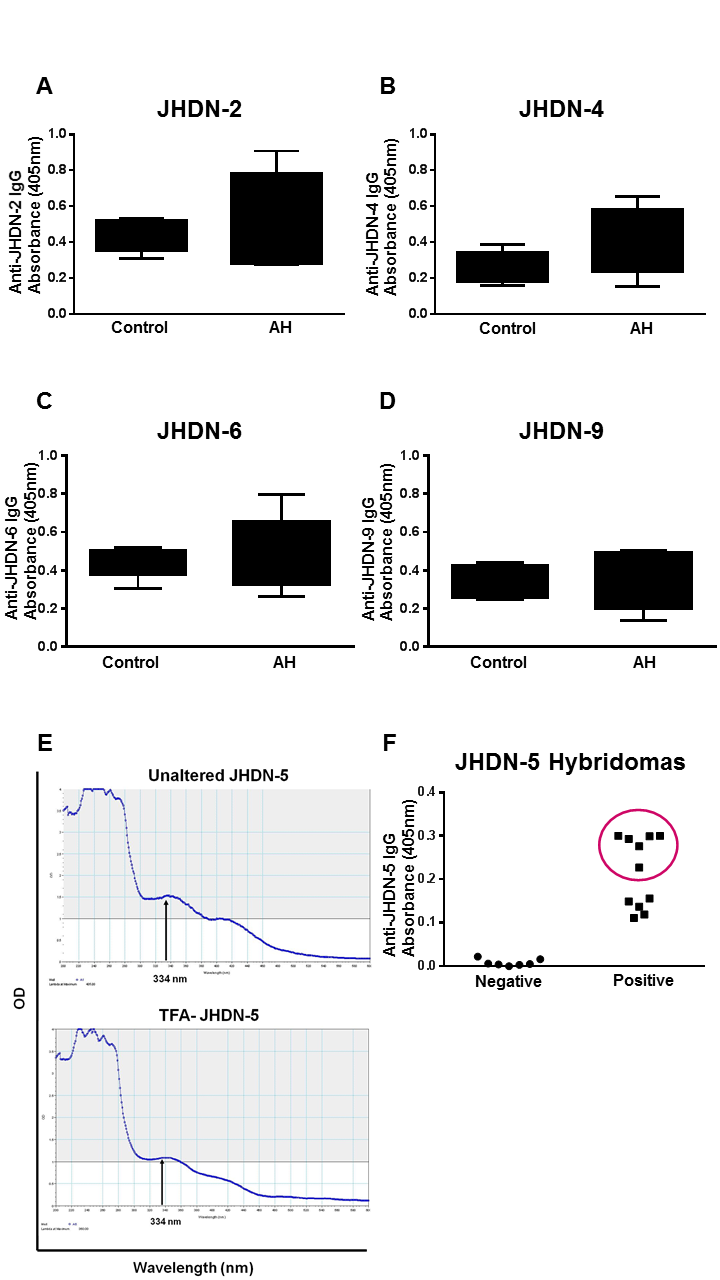

Supplement: FIG S1 [file sph005182659sf1.tif]

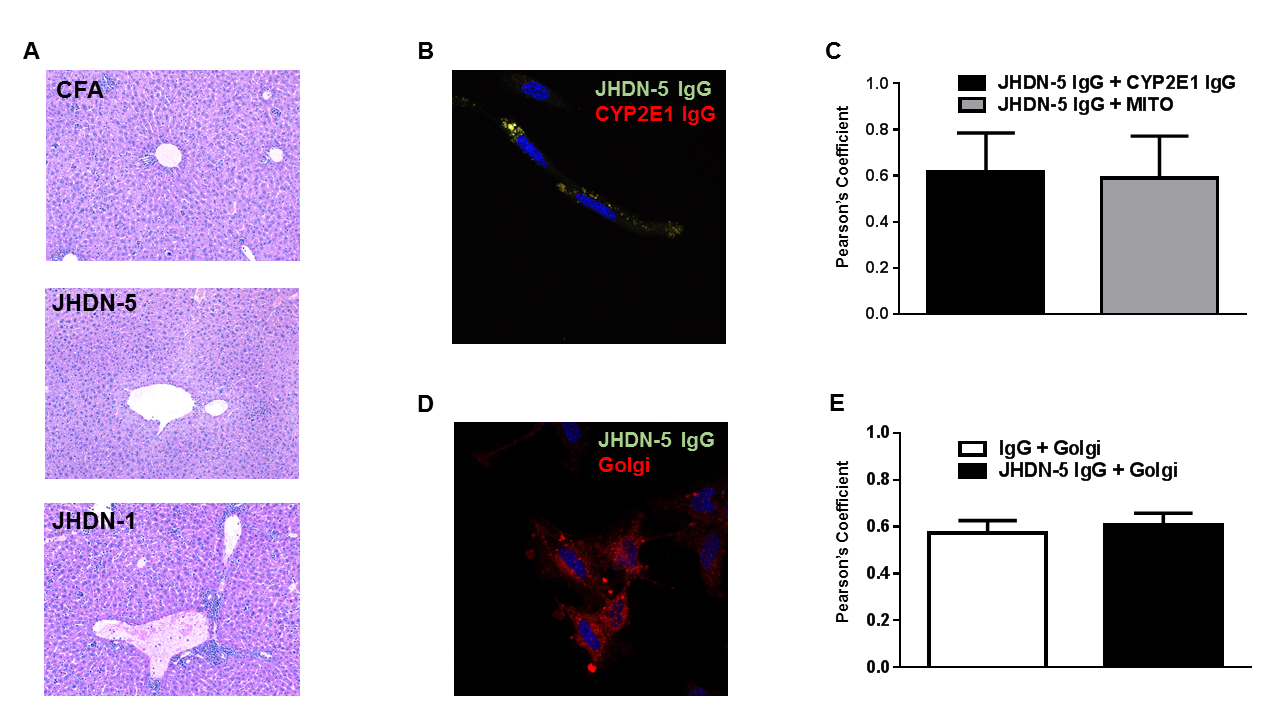

Supplement: FIG S2 [file sph005182659sf2.tif]

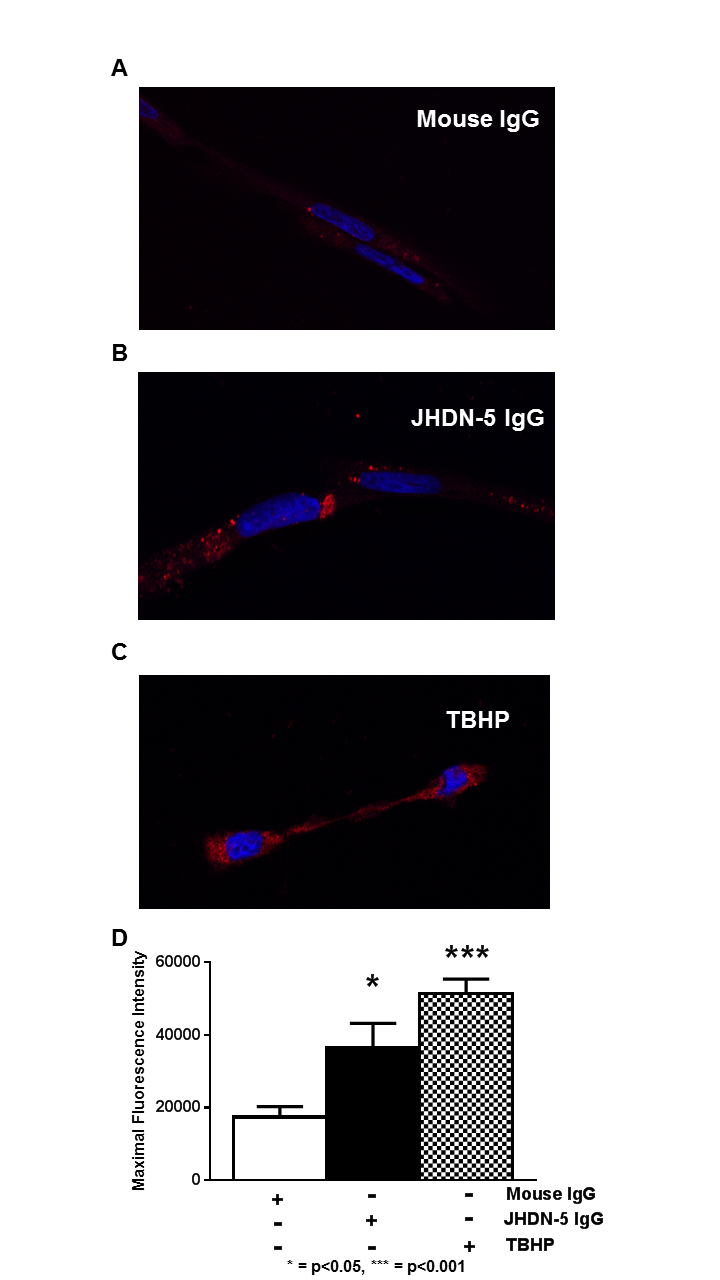

Supplement: FIG S3 [file sph005182659sf3.tif]

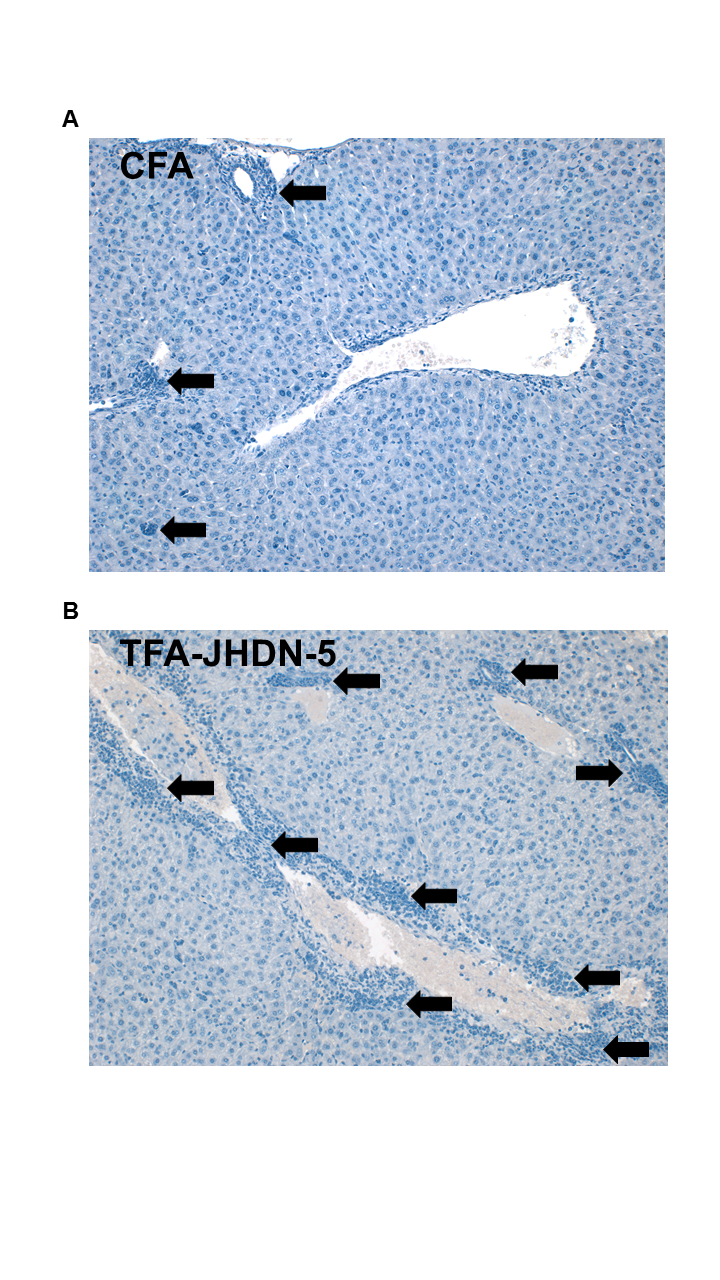

Supplement: FIG S4 [file sph005182659sf4.tif]
